# Band Gap Tuning of Films of Undoped ZnO Nanocrystals by Removal of Surface Groups

**DOI:** 10.3390/nano12030565

**Published:** 2022-02-07

**Authors:** Chengjian Zhang, Qiaomiao Tu, Lorraine F. Francis, Uwe R. Kortshagen

**Affiliations:** 1 Department of Chemical Engineering and Materials Science, University of Minnesota, Minneapolis, MN 55414, USA; zhan5641@umn.edu (C.Z.); tu000007@umn.edu (Q.T.); 2 Department of Mechanical Engineering, University of Minnesota, Minneapolis, MN 55414, USA

**Keywords:** metal oxide nanocrystals, band gap, Burstein–Moss effect, band gap renormalization, nonthermal plasmas, atomic layer deposition, intense pulsed light

## Abstract

Transparent conductive oxides (TCOs) are widely used in optoelectronic devices such as flat-panel displays and solar cells. A significant optical property of TCOs is their band gap, which determines the spectral range of the transparency of the material. In this study, a tunable band gap range from 3.35 eV to 3.53 eV is achieved for zinc oxide (ZnO) nanocrystals (NCs) films synthesized by nonthermal plasmas through the removal of surface groups using atomic layer deposition (ALD) coating of Al_2_O_3_ and intense pulsed light (IPL) photo-doping. The Al_2_O_3_ coating is found to be necessary for band gap tuning, as it protects ZnO NCs from interactions with the ambient and prevents the formation of electron traps. With respect to the solar spectrum, the 0.18 eV band gap shift would allow ~4.1% more photons to pass through the transparent layer, for instance, into a CH_3_NH_3_PbX_3_ solar cell beneath. The mechanism of band gap tuning via photo-doping appears to be related to a combination of the Burstein–Moss (BM) and band gap renormalization (BGN) effects due to the significant number of electrons released from trap states after the removal of hydroxyl groups. The BM effect shifts the conduction band edge and enlarges the band gap, while the BGN effect narrows the band gap.

## 1. Introduction

Transparent conductive oxides (TCOs), which possess a high transmittance of visible light and large electrical conductivity, are necessary components in optoelectronic devices such as flat-panel displays and photovoltaic applications [1]. Recently, TCO nanocrystals (NCs) have gained interest due to their plasmonic resonance behavior and size-dependent properties as well as the availability of low-temperature NC-based processing routes [2,3]. Indium tin oxide (ITO) is the most widely used commercial TCO, however, the limited amount of indium and worldwide growing demand for TCOs has stimulated the study of alternative TCOs [4,5]. Zinc oxide (ZnO) is a promising candidate because of its wide direct band gap of 3.3 eV, excellent optical transparency, low toxicity, and global abundance of its constituent elements [6].

Although many methods have been proposed for fabricating ZnO NCs, it is challenging to produce films of intrinsic ZnO NCs with a high yield that meet the requirements of high carrier mobility and low electrical resistivity [6,7]. Herein, we use nonthermal plasmas, a low temperature gas-phase synthesis technique, to synthesize ZnO NCs without intentional impurity doping. Due to two unique features of the nonthermal plasma synthesis, particle charging and particle confinement, the produced ZnO NCs have both narrow size distributions and high NC yields [8,9,10]. Although as-deposited ZnO NCs films are not suitable for TCO application, previous work has shown that appropriate post-treatments can significantly enhance their electronic transport properties. Thimsen et al. [11] showed that after coating ZnO NC films with aluminum oxide (Al_2_O_3_) by atomic layer deposition (ALD), the electron mobility of ZnO NC films was greatly improved, reaching up to ~10 cm^2^/V-s. Another study by Greenberg et al. [12] demonstrated that by further applying intense pulsed light (IPL) to ALD-coated ZnO NCs, the carrier density remarkably increased due to the elimination of electron traps. These results indicate that ZnO NC films with a high electrical conductivity and carrier mobility can be achieved by ALD coating and IPL treatment.

Aside from the electrical conductivity, the other significant parameter for TCO NC films is their optical performance, specifically their transparency, which is quantified by the transmittance of incident light. The upper limit of the optical transparency window of TCO NCs is determined by their plasmon resonance frequencies, which are usually in the infrared (IR) range and beyond the working range of most optoelectronics [13], while the lower limit is directly related to the band gap of TCO NCs. Band gap tuning of ZnO is widely studied but limited to extrinsic doping with elements such as Al, Ga, and Sn; tuning is mainly attributed to the Burstein–Moss effect [14,15,16,17]. However, there are very few reports on the band gap tuning of naturally doped ZnO without intentional doping. Since the band gap is significant to the performance of optoelectronics, studying the tunability of the band gap of our conductive ZnO NCs as well as the mechanism is worthwhile.

In this work, we explore the optical performance and band gap of ZnO NCs synthesized by nonthermal plasmas. The absorbance spectrum of ALD-coated and IPL-treated ZnO NCs is studied from the ultraviolet (UV) to visible light range. We demonstrate that the band gap of ZnO NC films can be tuned via IPL-induced photo-doping. Moreover, the observed band gap behavior is consistent with the combined effects of the Burstein–Moss effect and band gap renormalization. 

## 2. Materials and Methods

### 2.1. Sample Preparation and Post Treatments

**Scheme 1:**
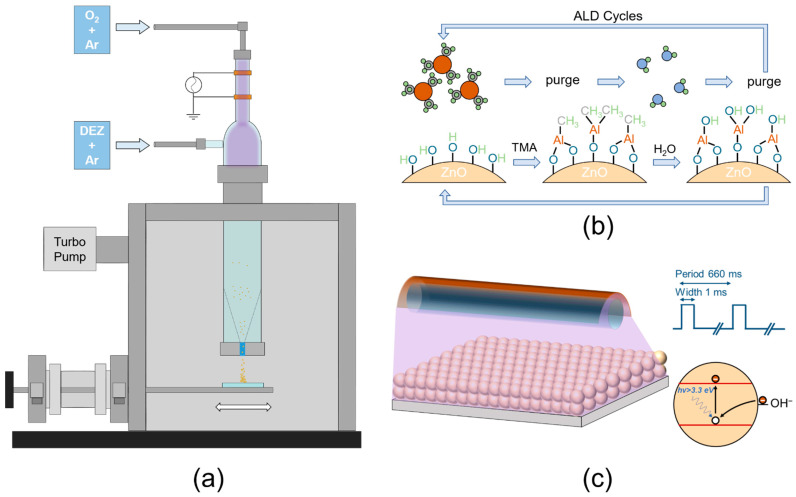
Schematics of (**a**) nonthermal plasma synthesis, (**b**) ALD, and (**c**) IPL.

ZnO NCs were synthesized in a reactor following the same procedure described in previous studies [11,12], as shown in Scheme 1a. In brief, the precursor diethylzinc (DEZ) and oxygen diluted in argon were flowed into a quartz tube where a nonthermal plasma was generated by applying radio frequency electric power to a pair of ring electrodes. ZnO NCs were formed through a sequence of steps, including precursor dissociation, cluster nucleation, and cluster agglomeration, to form NCs, with subsequent NC surface growth [8]. NCs were accelerated after passing through a nozzle and finally deposited onto 1 cm by 1 cm silicon or glass substrates by inertial impaction [18]. The substrates were moved back and forth 15 times to collect ZnO NC thin films. After collection was finished, the substrate with the ZnO NC thin film was removed from the reactor and exposed to air. These specimens are referred to as “as-deposited”.

The as-deposited ZnO NC films were coated with Al_2_O_3_ by ALD using a Cambridge Nanotech/Ultratech Savannah S2000 ALD reactor (Veeco Instruments Inc., Waltham, MA, USA) and an identical procedure to that described in a previous study [11]. During the process, Al_2_O_3_ was deposited onto the surfaces of ZnO NCs and thus infilled the voids between nanocrystals. The deposition took place at 180 °C by repeating cycles, each of which was comprised of four steps, as schematically shown in Scheme 1b. In the following, ALD-coated samples are labeled as “ALD” followed by the number of ALD cycles conducted (e.g., ALD-30).

Lastly, to achieve photo-doping, ALD-coated samples were placed into a Sinteron 2010 system (Xenon Corp., Wilmington, MA, USA) under ambient conditions, as shown in Scheme 1c. IPL treatments were performed using the same recipe as in ref. [12]. Briefly, photons with a broad wavelength range from 200 nm to 1000 nm were emitted from a xenon flash lamp in a pulse mode with a period of 660 ms and a pulse length of 1 ms. In the following, ALD-coated samples that were subsequently treated by IPL are labeled as “DIPL” followed by the number of IPL flashes (e.g., DIPL-10). Notably, all specimens labelled with DIPL were first coated with alumina in 70 ALD cycles. To understand the impact of the ALD coating, additional samples were only treated with IPL without ALD coating. These samples are labeled as “IPL” (e.g., IPL-10).

### 2.2. Materials Characterization

Two-dimensional X-ray diffraction (XRD) patterns were obtained from samples on silicon substrates using a Bruker D8 Discover diffractometer system (Bruker Corp., Billerica, MA, USA) with a cobalt source and a beryllium area detector. Fourier-transform infrared (FTIR) spectra were acquired from samples on the aluminum-coated silicon substrates using a Bruker Alpha IR spectrometer (Bruker Corp., Billerica, MA, USA) in diffuse reflectance mode under a nitrogen atmosphere. Ultraviolet–visible (UV–Vis) absorbance measurements were carried out on samples deposited on Corning Eagle XG glass substrates using a Cary 7000 UV-Vis spectrometer (Agilent Technologies Inc., Santa Clara, CA, USA). Single-wavelength ellipsometry at 632.8 nm was performed using a Gaertner (LSE Stokes) ellipsometer (Gaertner Scientific Corp., Skokie, IL, USA) on samples deposited on silicon substrates with ALD coating only to obtain the ALD coating thickness. The samples at different process stages were independent of each other and were measured separately.

## 3. Results

### 3.1. Characterization of ZnO NC Thin Films

As-deposited ZnO NC thin films have a morphology consisting of a direct-contact nanocrystal network with porosities in the range of 60–70% measured by ellipsometry (see Figure S1 in Supplementary Materials). The film thickness of samples in this study was around 150 nm, as determined by ellipsometry and confirmed by profilometry. Such thin and porous films provide a large three-dimensional surface for ALD deposition, enabling the efficient coating of Al_2_O_3_. The ALD coating thickness is 7.7 nm according to the single-wavelength ellipsometry measurement, which is sufficient to fill all pores insides the NC films, as demonstrated in ref. [11]. 

**Figure 1:**
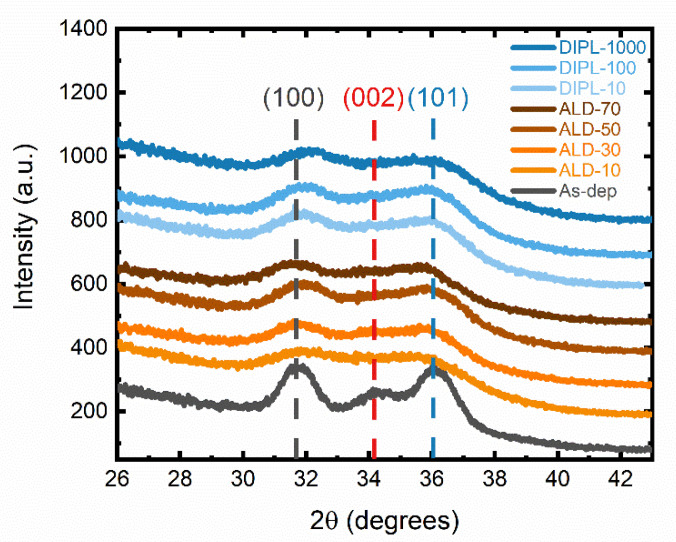
XRD patterns of as-deposited ZnO NCs; ZnO NCs after 10, 30, 50, or 70 cycles of ALD (ALD); and ZnO NCs after 70 cycles of ALD and 10, 100, or 1000 flashes of doping IPL (DIPL).

XRD patterns of ZnO NC films at different process stages are shown in Figure 1. The weak peaks or low signal-to-noise ratios are due to their thin film thickness, high porosity, and small crystallite size. The XRD pattern of as-deposited samples agrees with ICDD PDF #36-1451, suggesting that the ZnO NCs have a wurtzite structure. The average crystallite size is around 7.5 nm, calculated from (101) peak width by the Scherrer equation [19]. No extra peaks were observed for the XRD of ALD-treated samples, indicating that Al_2_O_3_ coating is amorphous, consistent with previous work [20]. The weaker peak intensities obtained for the films after ALD and IPL treatments can be ascribed to the scattering of X-rays from the amorphous Al_2_O_3_ coating. The peak widths of ALD and IPL samples are similar to those of the as-deposited samples, confirming that no crystal growth takes place during the post treatments. Potential strain effects were analyzed based on XRD peak shifts (see Note S1 and Figure S2 in Supplementary Materials) and are further discussed in a later section.

### 3.2. Band Gap of ZnO NC Thin Films

**Figure 2:**
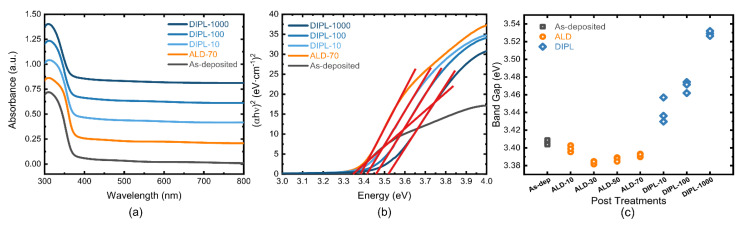
(**a**) UV–visible spectra of as-deposited ZnO NCs, ZnO NCs after 70 cycles of ALD, and ZnO NCs after 70 cycles of ALD and different number of doping IPL flashes; (**b**) corresponding Tauc plots and (**c**) corresponding band gaps, including ZnO NCs after 10, 30, and 50 cycles of ALD.

The UV–Vis spectra of the ZnO NC specimens are shown in Figure 2a. No extra spectral features for ALD and IPL samples were observed in the UV–Vis absorbance spectra, demonstrating that Al_2_O_3_ coating does not contribute to absorbance in this range, as expected based on the large band gap (6.2 eV) of amorphous Al_2_O_3_ [21]. However, we did observe shifts in the absorbance edge in the ultraviolet range. While these are difficult to see in Figure 2a, they appear more clearly in the Tauc plot in Figure 2b. The Tauc plot, a transformation the of UV–Vis spectrum, was prepared by plotting ${\left(\alpha h\nu \right)}^{n}$ versus the photon energy, where α is the absorption coefficient, hν is the photon energy, and *n* is an exponent that equals 2 for a direct band gap. This is a widely used method for determining the band gap of semiconductors (for calculations, see Note S2 in Supplementary Materials) [22]. The intercept of the red line representing the linear regime of the Tauc plot and the abscissa in Figure 2b is the band gap. The shifts in the intercept in these plots apparently show changes in the band gap.

The band gaps for samples at all processing stages extracted using the Tauc plots are shown in Figure 2c, revealing an interesting trend of band gap changes. The band gap of as-deposited ZnO NCs decreased by about 0.05 eV after ALD coating and remained almost unchanged as more ALD cycles were performed. However, the band gap of the ALD-treated samples increased after the IPL treatments and the amount of band gap shift depended on the number of IPL flashes. Overall, the band gap of the ZnO NC films varied from 3.38 eV to 3.53 eV, with a span of 0.15 eV. It is worth noting that as-deposited ZnO NCs had a band gap of 3.41 eV, which was close to that of bulk ZnO, 3.37 eV [23]. This result is reasonable because the size of our ZnO NCs was around 7.5 nm and much larger than the Bohr exciton radius of ZnO, 1.8 nm; hence, quantum confinement is not expected [24]. Clearly, the combination of ALD coating and IPL treatment is capable of tuning the band gap of ZnO NCs.

Apart from these results, the band gap of as-deposited ZnO dropped from 3.41 eV to 3.35 eV after 60 days of exposure to air, while the ALD-treated samples maintained a constant band gap (see Figure S3 in Supplementary Materials). In view of the hygroscopic nature of ZnO and the large surface areas of NCs, water vapor from the air likely absorbed on the ZnO NCs and thus changed the concentration of the surface groups. Hence, we hypothesize that the surface groups of ZnO NCs influence the band gap. This hypothesis motivated the study of the NC surface chemistry in the next section.

### 3.3. FTIR Study of ZnO NC Thin Films

**Figure 3:**
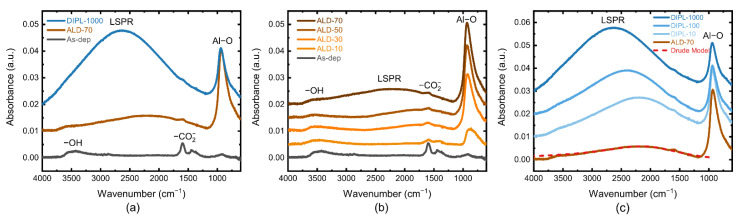
FTIR spectra of (**a**) as-deposited ZnO NCs, ZnO NCs after 70 cycles of ALD, ZnO NCs after 70 cycles of ALD and 1000 flashes of doping IPL (DIPL); (**b**) ZnO NCs after ALD with different numbers of cycles; (**c**) ZnO NCs after 70 cycles of ALD and DIPL with different numbers of flashes.

The FTIR spectra shown in Figure 3 provide information about the surface chemistry of the ZnO NCs and their changes after ALD coating and IPL treatments. Figure 3a shows an overview of the surface groups on as-deposited ZnO NCs and their changes based on post-processing steps. There are mainly two surface groups in as-deposited ZnO NCs: hydroxyl groups, −OH, and carboxylate groups, −COO^−^. These two groups are partly removed by ALD and the rest of the hydroxyl groups are further eliminated by IPL. The Al-O stretch observed in ALD-70 samples is a signature of the Al_2_O_3_ coating. A large broad peak is observed in the IPL-1000 sample, caused by a localized surface plasmon resonance (LSPR). An LSPR occurs when the free electrons in NCs oscillate coherently in response to the incident light and strongly absorb the light near the resonant frequency [25]; hence, the appearance of an LSPR peak indicates a large concentration of free carriers in IPL-treated samples. The free electrons likely originate from intrinsic defects such as oxygen vacancies and interstitial zinc [26,27,28]. These electrons are localized on surface hydroxyl groups on the as-deposited ZnO NCs and will be released when surface trap hydroxyl groups are removed under photonic treatment [11]. A schematic illustration of this process is shown in Scheme 1c. 

Additional results for the ALD- and IPL-treated samples are shown in Figure 3b,c. According to Figure 3b, the surface groups, −OH and −COO^−^, were largely reduced after the first few ALD cycles and additional ALD cycles only slowly removed additional surface groups on the ZnO NCs. A weak LSPR peak was observed in the ALD-70 sample, indicating that the electron density is large enough to support an LSPR. As demonstrated by Figure 3c, IPL effectively removed the hydroxyl groups remaining after ALD and released additional electrons. The peak corresponding to hydroxyl groups became smaller and smaller until it vanished as the number of IPL flashes increased, accompanied by the blueshift and enhancement of the LSPR peak. Since the LSPR resonant frequency is related to the electron density, the blueshift of the LSPR peak indicated an increase in the carrier density, as discussed later. The FTIR spectra also show that there are always small amounts of residual carboxylate groups, indicated by the carbon-oxygen stretch, regardless of the treatment of the ZnO NC samples.

**Figure 4:**
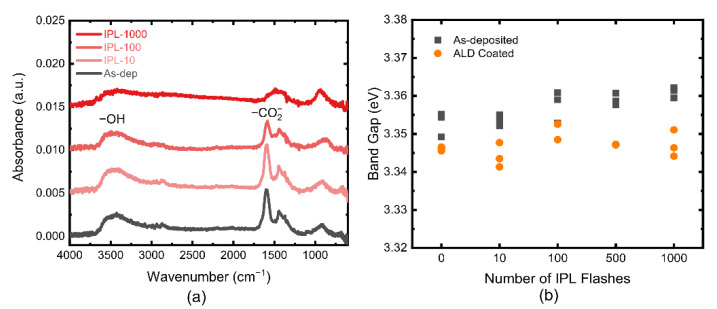
(**a**) FTIR spectra of IPL-treated ZnO NCs that were not coated by ALD before being subjected to 10, 100, or 1000 IPL flashes; (**b**) band gap of IPL-treated ZnO NCs with and without ALD coating after IPL treatment.

To better understand the effect of ALD and IPL on surface groups, additional FTIR experiments were carried out; see Figure 4a. In this set, only IPL without prior ALD was used. As-deposited ZnO NC films deposited on silicon substrates were directly placed under the xenon flash lamp after collection and treated with different numbers of IPL flashes. The FTIR spectra show that IPL removed the carboxylate groups of bare ZnO NCs, as ALD does, and that hydroxyl groups still persisted after 1000 flashes of IPL even though the OH absorption feature is reduced. However, since IPL was performed under ambient conditions and the samples were exposed to air prior to the FTIR measurements, new hydroxyl groups could be generated on the NC surfaces after IPL treatment. 

The band gaps of the samples mentioned in the last paragraph are plotted in Figure 4b. After determining the band gap of these ZnO NC films treated only by IPL, the samples were coated with Al_2_O_3_ by 70 cycles of ALD, denoted by IPL-ALD, and then the band gap was measured again. It can be seen that both the band gaps of as-deposited samples and IPL-ALD samples were independent of the number of IPL flashes, which demonstrates that the removal of the carboxylate groups was unrelated to the band gap variation. The difference in band gap between the as-deposited and IPL-ALD samples was small. Hence, band gap tuning requires ZnO NC films to be treated with ALD before IPL treatment. These results, together with the observation in Figure 2c, also indicate that band gap tuning requires the removal of a sufficient number of hydroxyl groups. When ALD is applied prior to IPL, the Al_2_O_3_ coating seals the ZnO NCs and protects them from reaction with water vapor in the ambient environment that would reform the hydroxyl groups. 

### 3.4. Band Gap Tuning Mechanism

Based on the above FTIR results, it appears that the occurrence of the LSPR, which indicates an increase in the free carrier density, is correlated to the removal of the hydroxyl groups. We hypothesize that the increase in the free carrier density also gives rise to increases in the band gap via the Burstein–Moss (BM) effect. The BM effect occurs when degenerate doping is achieved, which raises the Fermi level above the conduction band minimum, leading to an increase in the band gap [29,30]. A significant electron density is required for degenerate doping, which explains why the band gap shift occurs only after a critical degree of −OH removal is reached. 

**Figure 5:**
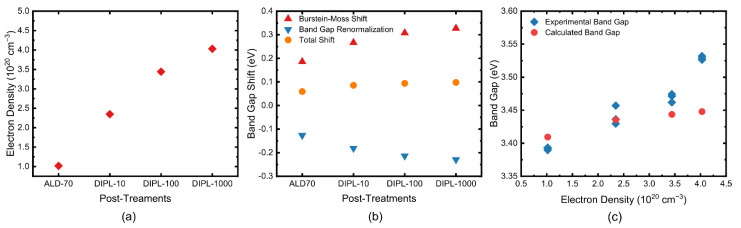
(**a**) Carrier density extracted from LSPR fitting, (**b**) calculation of Burstein–Moss shift and band gap renormalization, and (**c**) comparison of calculated and experimental band gaps.

To quantify whether the observed band gap shift is consistent with the BM effect, first the carrier density was quantified by fitting the LSPR peaks in FTIR spectra with a Drude model (see Note S3 in Supplemental Materials) [31,32]. One example fit is shown in Figure 3c. The electron densities extracted from the Drude model are shown in Figure 5a. Generally, the band gap shifts due to the BM effect can be related to electron density through the following equation [29,33]:

(1) $$\begin{array}{c} \Delta E^{BM}=\frac{h^{2}}{8m^{*}}{\left(\frac{3n}{\pi }\right)}^{\frac{2}{3}}, \end{array}$$

where n is the carrier concentration, h is Planck’s constant, and $m^{*}$ is the effective mass. However, this equation assumes a parabolic conduction band edge. In our case, NCs were heavily doped and a nonparabolic conduction band should be taken into account, which is related to a carrier concentration-dependent effective mass [34]. The effective mass can be calculated using the Pisarkiewicz model [35] and Equation (1) can be modified to [36]:

(2) $$\begin{array}{c} \Delta E^{BM}=\frac{h^{2}}{8m_{0}^{*}(1\;+\;0.5\left(m^{*}/m_{0}^{*}\;-\;1\right)}{\left(\frac{3n}{\pi }\right)}^{\frac{2}{3}}, \end{array}$$

where $m_{0}^{*}$ is the effective mass at conduction band minimum and $m^{*}$ is the effective mass at a certain carrier concentration. The calculated results are shown in Figure 5b.

However, a band gap narrowing (BGN) counteracting the BM effect is induced when a critical carrier concentration is reached, which is due to the many-body renormalization effect [37]. A consensus regarding the critical concentration for ZnO has not been reached. Ranges from $2\times 10^{19}$ to $8.4\times 10^{19}$ cm^−3^ for the onset of BGN have been reported [34,38,39]. The electron density in our case is in the range where BGN may be important. Initial studies suggested an empirical *n*^1/3^ dependence derived from the exchange interactions [37] and screening [40,41]; however, it was pointed out that this empirical law is not valid for intrinsic doped n-type ZnO [38]. Another equation of the form $An^{1/3}+Bn^{1/4}+Cn^{1/2}$ was proposed to predict BGN [42]:

(3) $$\begin{array}{c} \Delta E^{BGN}=\frac{1.83}{r_{S}}\frac{\Lambda }{N_{b}^{\frac{1}{3}}}R+\frac{0.95}{r_{S}^{\frac{3}{4}}}R+\frac{\pi }{2}\frac{1}{r_{S}^{\frac{3}{2}}N_{b}}\left(1+\frac{m_{min}^{*}}{m_{maj}^{*}}\right)R, \end{array}$$

where Λ is a correction factor accounting for band anisotropy; $N_{b}$ is the number of equivalent band extrema; R is the effective Rydberg energy for a carrier bound to a dopant atom; and $m_{maj}^{*}$ and $m_{min}^{*}$ are the majority and minority carrier density-of-state effective mass, respectively. $r_{S}$ is the average distance between majority carriers normalized to the effective Bohr radius a and can be calculated using the following equation:

(4) $$\begin{array}{c} r_{S}=\frac{{\left(\frac{3}{4\pi n}\right)}^{\frac{1}{3}}}{a}. \end{array}$$

Equation (3) was applied to ZnO, and good agreements between the theoretical and the experiment data have been reported [17,34]. Our calculated results are shown in Figure 5b. Finally, the combined optical band gap can be expressed by:

(5) $$\begin{array}{c} E_{g}=E_{g0}+\Delta E^{BM}-\Delta E^{BGN}, \end{array}$$

A comparison between calculated and experimental results is shown in Figure 5c. The calculated data fit the experimental data at lower electron concentrations, but discrepancy exists at higher electron densities.

## 4. Discussion

According to the above results, the observed band gap variation stems from the change in electron density, which is caused by the removal of electron-trapping hydroxyl surface groups. However, there is still a discrepancy between the experimental and calculated results, as shown in Figure 5c. Due to the lack of understanding of the Rydberg energy and effective mass for intrinsically doped ZnO, the values for Al-doped ZnO were used here, which is one of the possible reasons for the discrepancy. Another possible reason is the effect of the strain. It is known that a compressive strain, especially a strain along the c-axis, results in an increase in the ZnO band gap because of the increased overlap of Zn 4s-O 2p and Zn 3d atomic wave functions [43,44]. The existence of tensile stress in Al_2_O_3_ films produced by ALD has been widely studied [20,45], indicating that the ZnO NCs could be compressed by the surrounding ALD coating. However, the effect of strain is ruled out in our case based on the lack of significant differences in the XRD patterns and the lack of correlation between strain and band gap (for more details, see Figure S2 and Note S1 in Supplementary Materials).

At last, the increased percentage of transmitted photons was calculated (see Note S4 in Supplementary Materials). A widening of the ZnO NC band gap from 3.35 eV to 3.53 eV corresponds to a wavelength shift from 372 nm to 351 nm. Assuming an AM 1.5 g spectrum of incident radiation and a CH_3_NH_3_PbX_3_ perovskite solar cell with a photoresponse range from 350 nm to 750 nm [46], the number of photons transmitted by the ZnO NC films increases by 4.1% due to the band gap widening discussed here.

## 5. Conclusions

The optical performance of as-deposited, ALD-coated, and IPL-treated ZnO NC films was studied. A tunable band gap range from 3.35 eV to 3.53 eV for ZnO NCs was found. We attributed the observed band gap widening to the combined and competing mechanisms of the Burstein–Moss effect and band gap renormalization. The small peak shifts of XRD patterns ruled out the effect of strain on band gap. The FTIR spectra indicated that the ALD and IPL effectively removed the surface groups including hydroxyl groups and carboxylate groups. Upon the removal of hydroxyl groups, trapped electrons were released, contributing to an increased carrier density that we propose caused the Burstein–Moss effect and band gap renormalization when the concentration reached critical values. It was also demonstrated that the ALD coating was necessary for the band gap tuning, as the coating sealed the NCs and protected them from interaction with the ambient atmosphere, which would lead to the reformation of electron-trapping surface groups. This study provides an example of how to tune the band gap of ZnO NCs by the removal of surface groups using ALD coating and light exposure technology to increase the light transmission of ZnO NC films. It indicates the potential to apply this methodology to other TCO NC materials that are affected by electron-trapping surface groups.

## Data Availability

The data presented in this study are available on request from the corresponding author.

## Supplementary Materials

The following supporting information can be downloaded at https://www.mdpi.com/article/10.3390/nano12030565/s1: Figure S1: Structure characterization of as-deposited ZnO NCs: (a) Scanning electron microscope (SEM) image, (b) selected spectroscopic ellipsometry data, and (c) selected profilometry data. Figure S2: (a) XRD patterns of as-deposited and ALD-coated and IPL-treated ZnO, converted to Cu source, (b) peak position, and (c) linear strain: Band gap of as-deposited and ALD- and IPL-treated samples. Figure S3: Band gap of as-deposited, ALD and IPL-treated samples. Red and blue data points at each stage refer to the same samples measured right after synthesis and post-treatment (as-prepared) and 60 days later (60-day air exposure). The number after ALD and IPL indicates the number of cycles and number of flashes, respectively. Note S1: Strain analysis from XRD patterns and effect of strain. Note S2: Tauc method. Note S3: Estimation of the carrier density by Drude model. Note S4: Estimation of fraction of transmitted photons.
