# Supplementary material for: Band Gap Tuning of Films of Undoped ZnO Nanocrystals by Removal of Surface Groups
Source: Nanomaterials (Basel). 2022 Feb 7;12(3):565. doi: 10.3390/nano12030565 (PMC8838492; doi:10.3390/nano12030565)
Supplement: Supplementary file 1 [file nanomaterials-12-00565-s001.zip › nanomaterials-1561450/supplementary.pdf]

nanomaterials

*Supplementary Materials*

# Band Gap Tuning of Films of Undoped ZnO Nanocrystals by Removal of Surface Groups

**Chengjian Zhang [1], Qiaomiao Tu [1], Lorraine F. Francis [1,*] and Uwe R. Kortshagen [2,*]**

[1] Department of Chemical Engineering and Materials Science, University of Minnesota, Minneapolis, MN 55414, USA; zhan5641@umn.edu (C.Z.); tu000007@umn.edu (Q.T.)
[2] Department of Mechanical Engineering, University of Minnesota, Minneapolis, MN 55414, USA
* Correspondence: lfrancis@umn.edu (L.F.F.); kortshagen@umn.edu (U.R.K.)

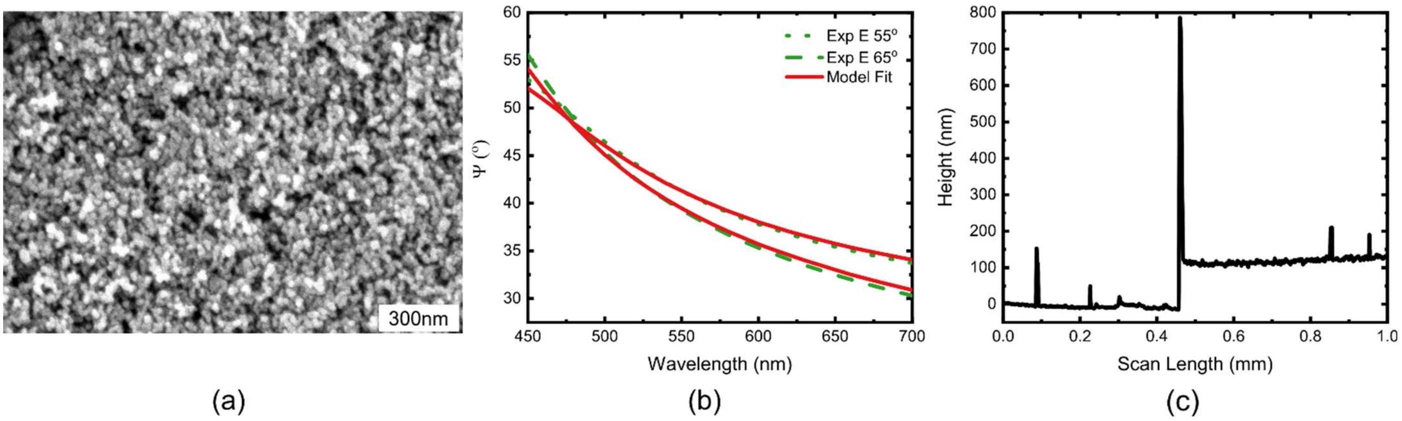


**Figure S1.** Structure characterization of as-deposited ZnO NCs: (**a**) Scanning electron microscope (SEM) image, (**b**) selected spectroscopic ellipsometry data and (**c**) selected profilometry data. SEM image was taken from a sample on the silicon substrate using a JEOL 6500 field emission gun (JEOL Ltd., Peabody, MA, USA). Spectroscopic ellipsometry data were carried out on samples on silicon substrates using a VASE spectroscopic ellipsometer (J.A. Woollam Co., Lincoln, NE, USA) and were fitted using effective medium approximations (EMA) model to obtain thickness and porosities. Profilometry was performed on samples on silicon substrates using a P16-surface profiler (KLA Corp., Milpitas, CA, USA).

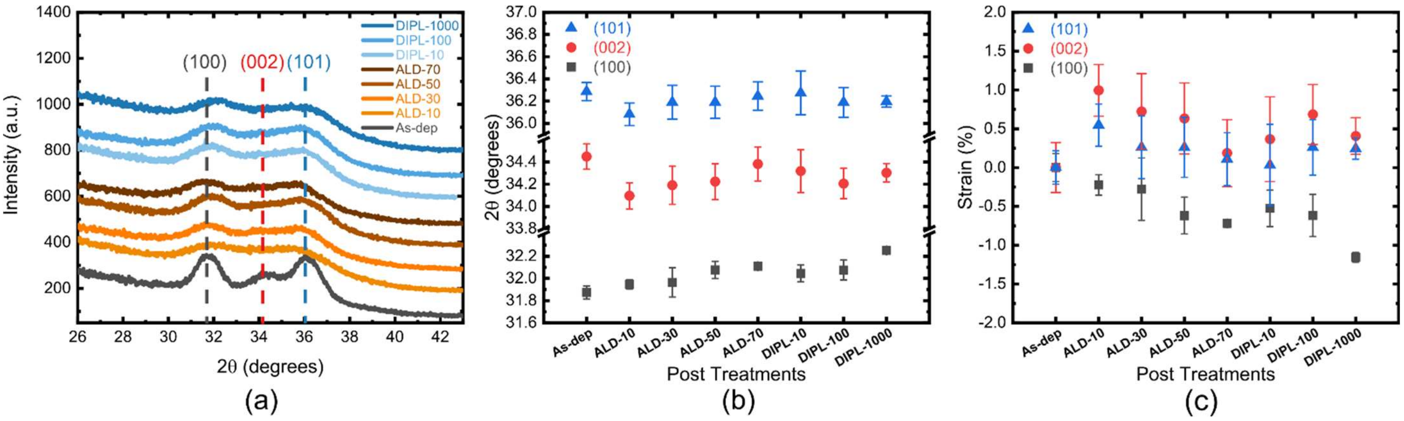


**Figure S2.** (**a**) XRD patterns of as-deposited, ALD coated and IPL treated ZnO, converted to Cu source, (**b**) peak position, and (**c**) linear strain. Error bars represent standard errors. The number after ALD and IPL indicates the number of cycles and number of flashes, respectively. See Note S1 for details.

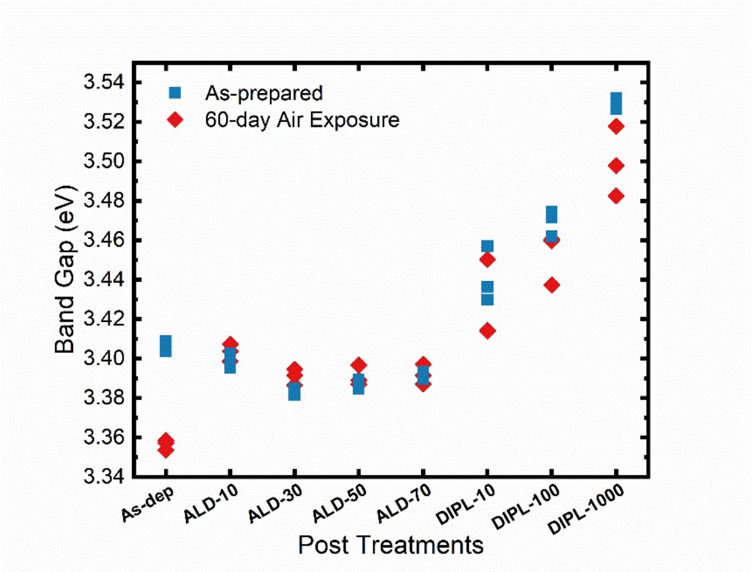


**Figure S3.** Band gap of as-deposited, ALD and IPL-treated samples. Red and blue data points at each stage refer to the same samples measured right after synthesis and post-treatment (as-prepared) and 60 days later (60-day air exposure). The number after ALD and IPL indicates the number of cycles and number of flashes, respectively.

## Note S1. Strain analysis from XRD patterns and effect of strain

The extraction of strain is based on the peak shift of XRD pattern. We define the peak shift as the difference between peak of a sample and that of as-deposited ZnO NC film sample, which is assumed to be strain-free. The ALD-10 sample is chosen for sample calculation. We start with the Gaussian fitting of the XRD pattern. According to this fitting, the (100) peak of ALD-10 sample is at 37.034°. The as-deposited sample is assumed to be strain-free and the (100) peak appears at 37.013°. The strain along the normal direction of (100) plane is calculated based on the Bragg's law as follow:

$$\varepsilon_{(100)} = \frac{\Delta d}{d_0} = \frac{d_1}{d_0} - 1 = \frac{\dfrac{\lambda}{2\sin\theta_1}}{\dfrac{\lambda}{2\sin\theta_0}} - 1 = \frac{\sin\theta_0}{\sin\theta_1} - 1 = \frac{\sin\dfrac{37.013^o}{2}}{\sin\dfrac{37.034^o}{2}} - 1 \approx -0.06\%, \quad (1)$$

where the subscripts 1 and 0 denote the ALD-10 and the as-deposited samples, respectively.

All peak positions, and linear strains extracted from XRD patterns are shown in Figure S2b and c. The linear strains of these samples between (002) planes are tensile and are supposed to lower the band gap of ZnO NCs [2,3]. However, the band gap remains almost unchanged throughout these stages as shown in Figure 2c, even if the strain changes. Furthermore, the standard errors are so large that error bars are overlapping with each other especially among ALD-50, ALD-70 and DIPL samples, indicating a lack of significant differences. Therefore, strain is not a significant factor for the band gap variation discussed here.

## Note S2. Tauc method

The Tauc plot or Tauc method is widely used to determine the band gap of a semiconductor [4]. The basic principle of the Tauc plot is that the absorption coefficient $\alpha$ is energy-dependent and can be expressed by the following equation:

$$(\alpha \cdot h\nu)^n = B\left(h\nu - E_g\right), \quad (2)$$

where $h$ is the Planck constant, $\nu$ is the photon's frequency, $E_g$ is the band gap energy, $B$ is a constant and $n$ is an exponent that is dependent on the nature of the electron optical

transition and is equal to 2 and 0.5 for the direct and indirect band gaps, respectively [5]. The exponent of 2 is used here as ZnO has direct band gap. According to equation (2), when $(\alpha \cdot h\nu)^n$ is plotted against $h\nu$ in the so called Tauc plot, the intercept with the abscissa will be the band gap $E_g$.

In order to obtain the Tauc plot, the sample spectral transmittance was measured using a Cary 7000 spectrophotometer and converted to the absorbance using Beer – Lambert law, described in equation (3). Here, zero reflectance is assumed. The absorption coefficient was computed from equation (4).

$$A_{abs} = -\log T, \tag{3}$$

$$\alpha = \frac{A_{abs}}{d} \times \ln 10 = \frac{2.303 \cdot A_{abs}}{d}, \tag{4}$$

where $A_{abs}$, $T$, $\alpha$ and $d$ refer to absorbance, transmittance, absorption coefficient and film thickness, respectively. The band gap $E_g$ is extracted from the linear region of the $(\alpha \cdot h\nu)^n$ curve extrapolated to the intercept with the abscissa.

**Note S3. Estimation of the carrier density by Drude model**

The electron density $n$ is quantified by fitting LSPR absorption features measured by FTIR. The overall effective dielectric function of the film, $\varepsilon$, is estimated by Maxwell Garnett effective medium approximation (MG EMA):

$$\frac{\varepsilon - \varepsilon_m}{\varepsilon + 2\varepsilon_m} = \frac{\phi(\varepsilon_{NC}(\omega) - \varepsilon_m)}{\varepsilon_{NC}(\omega) + 2\varepsilon_m}, \tag{5}$$

where $\phi$ is the nanocrystal (NC) volume fraction, $\varepsilon_m$ is the dielectric constant of the medium ($Al_2O_3$ in this case, $\varepsilon_m = 2.6$), and $\varepsilon_{NC}(\omega)$ is the frequency-dependent dielectric function of NCs, which is given by the Drude equation:

$$\varepsilon_{NC}(\omega) = \varepsilon_\infty - \frac{\omega_p^2}{\omega^2 + i\omega\Gamma}, \tag{6}$$

where $\varepsilon_\infty$ is the high-frequency dielectric constant (3.7 for ZnO) and $\Gamma$ is the carrier relaxation frequency which is given by:

$$\Gamma = \frac{e}{\mu_l m^*}, \tag{7}$$

where $e$ is the elementary charge, $\mu_l$ is the local electron mobility, and $m^*$ is electron effective mass ($0.3m_e$ for ZnO). The plasma frequency $\omega_p$ is given by:

$$\omega_p^2 = \frac{ne^2}{\varepsilon_0 m^*}, \tag{8}$$

where $\varepsilon_0$ is the permittivity of the free space. Finally, the value of electron density n is obtained by fitting the spectrum to imaginary part of ε.

**Note S4. Estimation of the fraction of transmitted photons**

The solar irradiation spectrum is simulated by 5800K blackbody radiation. The calculation is based on Planck formula, which has the following form:

$$B_\nu = \frac{2h\nu^3}{c^2} \frac{1}{e^{\frac{h\nu}{k_B T}} - 1}, \tag{9}$$

where $B_\nu$ is the spectral radiance of a black body per unit frequency, $c$ is the light speed, $h$ is Planck constant, $k_B$ is the Boltzmann constant, $\nu$ is the frequency and $T$ is the temperature. Perovskite, $CH_3NH_3PbX_3$, is assumed as the solar cell material which has a sensitive wavelength range from 350 nm to 750 nm, corresponding to a energy range from 3.54 eV to 1.65 eV [6]. The TCO layer is the ZnO NC film with a variable band gap from 3.35 eV

and 3.53 eV, corresponding to a wavelength shift from 371 nm to 352 nm. The fraction of the blackbody radiation that can be absorbed by the silicon solar cell is calculated as follow:

$$F_{3.35} = \frac{\int_{1.65eV}^{3.35eV} B_\nu d\nu}{\int_0^\infty B_\nu d\nu} \times 100\% = 44.89\%, \quad (10)$$

$$F_{3.53} = \frac{\int_{1.65eV}^{3.53eV} B_\nu d\nu}{\int_0^\infty B_\nu d\nu} \times 100\% = 46.75\%. \quad (11)$$

Hence, widening the ZnO NC bandgap from 3.35 eV to 3.53 increases the amount of radiation than can be absorbed by $CH_3NH_3PbX_3$ perovskite solar cell:

$$\left(\frac{46.75\%}{44.89\%} - 1\right) \times 100\% = 4.1\%. \quad (12)$$

**References**


1. Lee, J.; Gao, W.; Li, Z.; Hodgson, M.; Metson, J.; Gong, H.; Pal, U. Sputtered deposited nanocrystalline ZnO films: A correlation between electrical, optical and microstructural properties. *Appl. Phys. A Mater. Sci. Process.* **2005**, *80*, 1641–1646, doi:10.1007/s00339-004-3197-6.
2. He, H.P.; Zhuge, F.; Ye, Z.Z.; Zhu, L.P.; Wang, F.Z.; Zhao, B.H.; Huang, J.Y. Strain and its effect on optical properties of Al-N codoped ZnO films. *J. Appl. Phys.* **2006**, *99*, doi:10.1063/1.2161419.
3. Tauc, J.; Grigorovici, R.; Vancu, A. Optical Properties and Electronic Structure of Amorphous Germanium. *Phys. status solidi* **1966**, *15*, 627–637, doi:https://doi.org/10.1002/pssb.19660150224.
4. Makuła, P.; Pacia, M.; Macyk, W. How to correctly determine the band gap energy of modified semiconductor photocatalysts based on UV–Vis spectra 2018.
5. Lu, H.; Tian, W.; Cao, F.; Ma, Y.; Gu, B.; Li, L. A self-powered and stable all-perovskite photodetector–solar cell nanosystem. *Adv. Funct. Mater.* **2016**, *26*, 1296–1302.
